# Embodying a Part-Time State Medical Service and State Insurance Scheme

**Published:** 1919-03-08

**Authors:** 


### A MINISTRY OF HEALTH PAMPHLET.

### Embodying a Part-Time State Medical Service and State Insurance Scheme.

The Editor has asked the writer to comment on a pamphlet with the above title, the author of which is Dr. H. E. C. Keith Murray. The pamphlet is, as the author says in the introduction, an outline. It is an outline of great width and breadth. One would like to see the details filled in. If this were done it would probably be found that the picture would be too crowded, that more than one canvas was necessary to deal with all the subjects. None the less, the outline is valuable. It shows the lines in which to build.

The formation of a Ministry of Health is one thing, what it shall do is another, and how it shall do it is a third. But, of course, the purpose governs the foundation, and the tools must be suited to the work to be done with them. Now it is not very difficult for an observant man with constructive imagination, the most valuable form of imagination, to make such an outline as this, but when it comes to working out the details the difficulties are immense. The author is wise in stipulating that all the functions of all the Health Authorities and Departments shall be brought under a common head. That is most desirable. Nohow else can overlapping in some places and friction in others be avoided. So the Ministry of Health is to be that head, and because it is to perform or guide the functions of all Health authorities by deciding what all those functions are and what others should be added to them, an idea may be obtained of the necessary constitution of the Ministry. Since it is to be concerned with health it is obvious that qualified medical men must be included in the Ministry and form a considerable percentage of its strength. But is a Medical Minister of Cabinet rank necessary? Presumably "medical" means a duly qualified medical man. This seems to narrow the choice of the man to be Minister of Health to an undesirable degree. A Minister must be in Parliament. It is not likely any Parliament will ever contain many qualified medical men, and, so far, it is seldom that men much considered by their professional brethren as very eminent professionally have succeeded as candidates. To limit the choice of Minister to one of half-a-dozen or a score of medical politicians is not desirable. There is no need that the Minister should himself be qualified. His chief duty is representation to Parliament of the health needs of the community. If he has an able staff and able advisers, many of both drawn from the ranks of qualified medical men, he will be well equipped if he be a capable man, to perform all his functions without necessarily being qualified himself.

It is, perhaps, a mistake to mix up assurance with health. It may be found better to separate the two completely. Every man and woman in the community should assure himself or herself against indigency due to sickness, accident, or old age, by regular payments from earnings. The amount of such contribution is a matter for actuaries. Any large assurance society would quote a weekly premium, commencing at any age, for a pension of any sum desired at any age required. If every person in Great Britain, irrespective of income or social position, was compelled or consented to take out at age eighteen years a policy for an old-age pension of £1 a week, to commence at age sixty-five years, the premium would be very small. Sickness maintenance pensions and accident maintenance pensions should each be treated as separate business propositions, and not mixed up with contributions to pay for proper treatment when out of health. That is yet another proposition, and properly comes under a Ministry of Health, for it is from such payments that the funds of the Ministry should come as to a great part. Both the question of assurance and the question of sick attendance contributions for death concern friendly societies. The pamphlet suggests that these societies should be absorbed by the State. This is a thorny suggestion. There are many strong arguments against it. It is an education in itself to be a member of a friendly society and to be interested in its affairs. That is an aspect of friendly societies that should never be lost sight of.

It is probably a mistake to fix an income limit above which people need not come into the scheme. The community is a community, and should act together as a community. It is a good lesson to do so. And if there be an income limit, it is certain to result in a division of the medical profession into two broad classes, the one those who will accept work under the State, the other that will not but will prefer to cater for the more wealthy. Probably the less irksome and the better paid work will be amongst the wealthy. The wealthy will pay the competent man of the better sort, but will refuse the rough and inefficient. The latter, therefore, will tend to go to State service, whilst the former will refuse it or leave it when they see a chance by so doing to gain a desirable practice. Amongst those who accept service there is suggested a division into whole-time men and part-time men. Between those also there is likely to be a cleavage.

Part-time work is seldom satisfactory. It is still true that a man cannot serve two masters. When an income is fixed and paid by the State, and another is capable of improvement and depends on personal exertion, the best of the men are likely always to go to the latter work more than to the former.

It is obviously impossible to comment in the space The Hospital can spare on all the outlines of the pamphlet, for it deals in twelve pages with enough subjects for as many Acts of Parliament, but it is well worth every medical man's while to get it and consider it. He cannot fail to get ideas from it, and will be greatly benefited in his understanding of the subjects if he takes paper and attempts to fill in the outlines and to draft schemes and bills.

